# HLA-G-specific microRNAs a novel approach for targeting tumors

**DOI:** 10.1186/2051-1426-1-S1-P174

**Published:** 2013-11-07

**Authors:** Simon Jasinski-Bergner, Arndt Hartmann, Verena Spath, Stefan Huettelmaier, Juliane Braun, Juergen Bukur, Barbara Seliger

**Affiliations:** 1 Martin Luther University Halle-Wittenberg, Institute of Medical Immunology, Halle, Germany; 2 University of Erlangen, Institute of Pathology, Erlangen, Germany; 3 Martin Luther University Halle-Wittenberg, Institute of Molecular Medicine, Halle, Germany

## 

Immune inhibitory molecules on immune cells as well as on tumor cells appear to play a key role in modulating the immunogenicity of tumors and the efficacy of immunotherapies. One mediator is represented by the non-classical HLA-G antigen, which is often overexpressed in human tumors when compared to corresponding normal tissues thereby inhibiting both innate as well as adaptive immune responses. Since discordant HLA-G transcript and protein expression levels were often found in tumors of distinct origin posttranscriptional control mechanisms have been recently suggested. Indeed, different HLA-G-specific miRs have been identified, which were able to downregulate HLA-G surface expression. The miR-mediated inhibition of HLA-G enhanced the NK cell recognition. These miRs were also differentially expressed in renal cell carcinoma (RCC) versus normal kidney epithelium. Immunohistochemical analysis demonstrated of a high frequency HLA-G expression in RCC lesions, which was associated with disease progression and inversely correlated with the expression of HLA-G-specific miRs. These data postulate that HLA-G-specific miRs might be used as prognostic markers as well as potential therapeutics for targeting HLA-G expressing RCC.

